# Swine manure application enriches the soil food web in corn and soybean production

**DOI:** 10.21307/jofnem-2019-014

**Published:** 2019-04-15

**Authors:** Zane J. Grabau, Yong Bao, Jeffrey A. Vetsch, Senyu Chen

**Affiliations:** 1 Entomology and Nematology Department, University of Florida, 1881 Natural Area Drive, Gainesville, FL, 32601; 2 Southern Research and Outreach Center, University of Minnesota, 35838 120th Street, Waseca, MN, 56093

**Keywords:** Nematode community, Corn, Soybean, Fertilizer, Manure

## Abstract

Strategies for managing plant-parasitic nematodes while promoting soil quality are needed in corn (*Zea mays*) and soybean (*Glycine max*) cropping systems. Therefore, a series of two-year experiments were conducted in Minnesota to determine the simple and interactive effects of manure or conventional fertilizer and short-term crop rotation on the nematode community, a sensitive indicator of soil ecology. The two-year crop sequences were Sus-Sus, Res-Sus, and Corn-Sus, where Sus and Res are soybean susceptible and resistant to *Heterodera glycines* (soybean cyst nematode: SCN), respectively. The fertilizer treatments were liquid swine manure, conventional phosphorus (P)-potassium (K) fertilizer, and no fertilizer. Crop sequence and fertilizer choice had individual main effects, but did not have an interactive effect on the nematode community. Swine manure affected the nematode community in ways that conventional PK fertilizer or no fertilizer did not, substantially enhancing populations of bacterivores in colonizer-persister group 1, which are extreme enrichment opportunists. Manure application did not affect other groups of free-living nematodes and decreased nematode community diversity. Conventional PK fertilizer did not influence the nematode community compared with untreated control. The effects of short-term crop sequences were much less pronounced and consistent than manure application, but corn altered the environment to favor fungivores while soybean increased bacterivore abundances.

In the United States, corn and soybean are among the most important crops, comprising 36.0 and 36.2 million hectares, respectively, in 2018, which was 55.5% of total area planted to principal crops (NASS-USDA, 2018). Since these crops cover such a large area, identifying management practices in corn and soybean production that promote sustainability (productivity over time while conserving natural resources) is an important goal. Nematodes play an important role in sustainability because management of plant-parasitic nematodes is necessary to optimize crop productivity (Grabau and Chen, 2016a, 2016b) and non-parasitic, free-living nematodes contribute to and are sensitive indicators of soil fertility and ecology (Bongers, 1990; Ferris et al., 2001).

In particular, soybean cyst nematode (*Heterodera glycines*) is the major yield-limiting pest in soybean production (Koenning and Wrather, 2010). Additional strategies to manage this pest are needed because management relies on a narrow set of practices including corn-soybean crop rotation (Grabau and Chen, 2016a, 2016b) and use of resistant cultivars (Chen et al., 2001), most of which are derived from a single parent source. Swine manure application is one alternative strategy as it has been shown to suppress soybean cyst nematode (SCN) through release of nematicidal compounds in greenhouse studies (Xiao et al., 2007, 2008). Additionally, swine manure and other fertilizers may improve crop production and tolerance to nematode infection by improving soil fertility and quality (Bao et al., 2013). In addition to physical and chemical components provided by fertilizers, biological components, such as free-living nematodes and the soil-dwelling organisms associated with them, are important contributors to soil nutrient cycling and quality. Fertilizer application may influence soil ecology and the nematode community because it provides an influx of nutrients and other compounds. Application of animal manures or plant-based fertilizers may influence soil ecology in a different manner than conventional fertilizers. Unlike conventional fertilizers, manures and plant-based fertilizers contain carbon sources that stimulate population growth of soil-dwelling organisms (Hernandez et al., 2007). When manures are applied, plant-available nutrients are released over multiple seasons and organic material may persist for an extended period of time (Diaz et al., 2012), so the residual impact of manure application on soil ecology over multiple seasons is of interest.

The influence of fertilizers on soil ecology in corn-soybean crop rotation systems is of interest because rotation with corn or SCN-resistant soybean is the main SCN management strategy and soil community response to fertilizers may vary by crop. In particular, both nutrient uptake (Halvorson and Schlegel, 2012) and root exudate profiles (Wagner and Broder, 1993) differ between corn and soybean, so nutrients and compounds available to the soil nematode and microbial community may vary by crop. Soil structure, plant residue volume and nutrient content, soil moisture, and other factors also vary between corn and soybean (Wagner and Broder, 1993; Nickel et al., 1995; Pedersen and Lauer, 2004; Halvorson and Schlegel, 2012) which may influence soil community responses to fertilizer application. Because of these factors, corn and soybean can also directly influence nematode community composition with corn stimulating fungivore population growth but soybean stimulating bacterivore population growth, particularly after multiple years of monoculture (Grabau and Chen, 2016c).

The long-term aim of this study was to improve sustainability of corn-soybean systems by identifying alternative strategies to suppress SCN, reduce crop damage from SCN, improve crop yield, and improve or maintain biological components of soil quality. Plant-parasitic nematode management and crop yield results from the study were reported in a previous paper (Bao et al., 2013). This paper primarily focuses on the influence of fertilizer application and crop rotation on components of soil quality. Previously reported studies in the region have investigated the impact of swine manure or conventional synthetic or mined nitrogen (N) – phosphorus (P) – potassium (K) – sulfur (S) fertilizer and nematicide application in combination with conventional or conservation tillage (Grabau et al., 2018) as well as long-term corn and SCN-susceptible soybean crop sequences crossed with nematicide application on the nematode community (Grabau and Chen, 2016c). The specific objectives of this study are to assess the simple and interactive effects of fertilizer application (swine manure and conventional PK fertilizer) and short-term crop sequences on the nematode community. Specific hypotheses included: (i) manure application provides carbon sources to the nematode community that conventional PK fertilizer or no fertilizer does not resulting in an enrichment of the nematode community; (ii) short-term crop sequences do not influence the soil environment enough to impact the nematode community within the two-year scope of this study; and (iii) short-term crop sequences and fertilizer application have an interaction effect on the nematode community, particularly that soybean cropping enhances the enrichment effects of swine manure.

## Materials and Methods

### Experimental design

Data for this study were collected from the same field experiments described in the study of Bao et al., (2013), and conducted from 2009 to 2010 in Waseca, MN. Experiments were conducted at sites with varying levels of SCN suppression (Bao et al., 2013). The sites were an SCN-suppressive site (S-site) where SCN abundances on SCN-susceptible soybean have been much less than average compared with similar fields in the region (Bao et al., 2011) and an SCN-conducive site (C-site). The level of suppressiveness at the two field sites has been previously assessed in greenhouse assays (Chen, 2007; Bao et al., 2011) and greenhouse tests demonstrated that suppressiveness to SCN at the S-site was partly due to soil microbes (Bao et al., 2011).

The S-site (44° 04′ 21″ N, 93° 31′ 24″ W) is a Nicollet clay loam (fine loamy, mixed, mesic Aquic Hapludoll). The C-site (44° 05′ 30″ N, 93° 32′ 47″ W) is a Webster clay loam (fine loamy, mixed, mesic Endoaquoll). At each site, the experiment was a randomized complete block design in a split-plot arrangement with four replicates. The main plot factor was crop sequence and the subplot factor was fertilizer. The crop sequence treatments were (i) SCN-susceptible soybean (Sus), (ii) SCN-resistant soybean (Res), or (iii) corn in 2009 followed by susceptible soybean in 2010. The fertilizer treatments were (i) liquid swine manure (manure), (ii) conventional P-K fertilizer (PK), and (iii) no fertilizer applied in 2009. The manure (37.4 m^3^ manure/ha or 239 kg total N/ha + 26 kg P/ha + 112 kg K/ha) was injected 10 cm under the soil at 76 cm spacing and crops were planted directly over the injected area in 2009. Conventional PK fertilizer was applied to the surface of the soil at 49 kg P/ha and 93 kg K/ha and incorporated with tillage before planting in 2009. Nitrogen was not included in the conventional fertilizer application because growers do not typically apply nitrogen to soybean, which fixes its own nitrogen. None of the plots received fertilizer in 2010. Conventional tillage practices were employed in the fall at both sites.

### Soil sampling and nematode community quantification

A composite soil sample was collected from each plot. For each sample, 20 soil cores were taken at 0 to 20 cm depth in the two central rows of the plot with a 2-cm-diameter soil probe. Plots were sampled at four different times: (i) before fertilizer application and planting, (ii) 45 d after planting (DAP) – in 2009 only, (iii) midseason – around two months after planting, and (iv) harvest. Soil was stored at 10 °C and processed for nematodes within 2 d.

Soil samples were mixed by manually pushing samples through a metal screen with 4 mm square apertures. Nematodes were extracted from a 100 cm^3^ soil subsample for each plot by hand-decanting and sucrose centrifugation (Jenkins, 1964). Subsequently, vermiform plant-parasitic and free-living nematodes were identified to genera morphologically by microscope and quantified. Abundances (nematodes/100 cm^3^ soil) of herbivores, bacterivores, fungivores, and omnivores/predators were calculated (Yeates et al., 1993). Herbivores consisted primarily of SCN and spiral nematodes, which have the potential to cause an economically important level of damage, as well as nematodes in the Suborder Tylenchinae or Family Psilenchidae, which are not thought to cause an economically important level of damage to crops. Abundances of nematode feeding guilds were also calculated, for use in select analyses, based on feeding groups and Bongers (1990) colonizer-persister (c-p) scale.

Various nematode community indices were also calculated based on abundances and ecological niches of nematodes in each plot. These indices included ratio of fungivore and bacterivores to herbivores (FBPP), Shannon–Weaver diversity index (Shannon, 1948), structure index (SI), channel index (CI), and enrichment index (EI). Briefly, the structure index measures the number of trophic links in the food web with higher values indicating a more structured food web (Ferris et al., 2001). The enrichment index measures food web enrichment based on the weighted relative abundance of colonizer nematodes (Ferris et al., 2001). The channel index (Ferris et al., 2001) measures fungal decomposition channels (greater values) relative to bacterial (lesser values). FBPP is an indicator of whether overall impact of the nematode community is positive or negative (Wasilewska, 1989).

### Statistical analysis

Data were analyzed separately for each season because treatment effects varied by season, but combined between sites. Dependent variables were evaluated for normality and homogeneity of variance and nematode abundances were transformed by natural log before analysis while nematode community indices were not transformed. A modified three-factor (site by crop sequence by fertilizer) ANOVA, for combining two split-plot experiments (Carmer et al., 1989), was conducted for each response variable. Site main effects were treated as random effects and were included as a source of variation, but not tested for significance (Carmer et al., 1989). Site was treated as a random factor, but site by crop sequence and fertilizer interactions were treated as fixed effects and tested for significance to determine if crop sequence and fertilizer effects were consistent from site to site. A level of *α* = 0.05 was used for determining significance in ANOVA models and separating fertilizer or crop sequence treatments using Fisher’s protected LSD. Data were analyzed using R version 3.0 (The R Foundation for Statistical Computing, Vienna, Austria).

## Results

### Trophic group abundances

**Figure 1:**
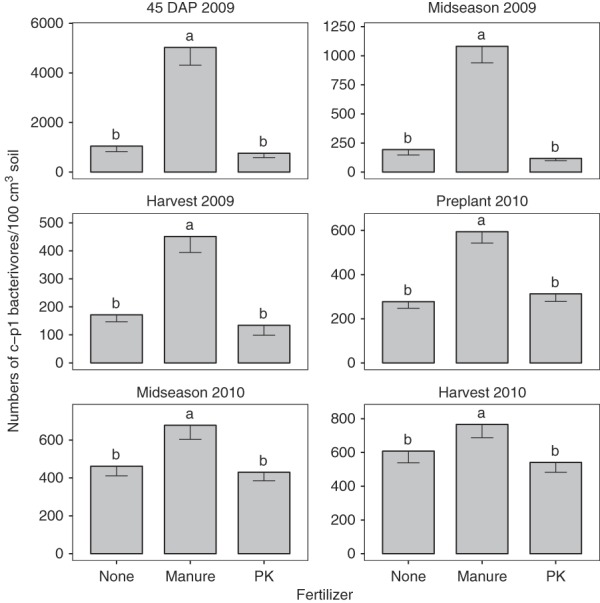
Colonizer-persister group 1 bacterivores as affected fertilizer treatments by sampling date combined across SCN-conducive and suppressive sites. Fertilizer treatment means with different letters in the same season are significantly different according to Fischer’s protected least significance test at *P* < 0.05.

**Figure 2:**
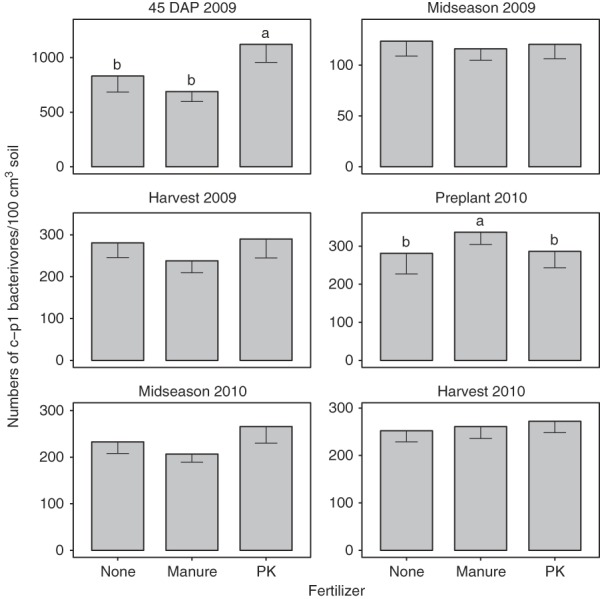
Colonizer-persister group 2 bacterivores as affected by fertilizer treatments combined across SCN-conducive and suppressive sites. Fertilizer treatment means with different letters in the same season are significantly different according to Fischer’s protected least significance test at *P* < 0.05.

Bacterivore abundances were significantly affected by crop sequence in Fall 2009 and Spring 2010 with abundances greater for resistant soybean than corn or susceptible soybean (Table 1). Manure consistently increased bacterivore abundances compared with no fertilizer or chemical fertilizers from 45 DAP 2009 through Midseason 2010 (Table 1). This trend was driven primarily by c-p1 bacterivores as manure consistently increased abundances of this group compared with no fertilizer or chemical fertilizers (Fig. 1), but generally did not affect c-p2 bacterivores (Fig. 2) and there were very few bacterivores belonging to any other guild.

**Table 1:** Effects of crop sequence and fertilizer on bacterivore abundances by sampling date combined across SCN-conducive and suppressive sites.^a^

	2009								2010				
	Pi	^b^	P45d		Pm		Pf		Pi		Pm		Pf
*Crop sequence*^*c*^													
Corn-Sus	1,077		3,056		601		309	C	545	B	743		962
Res-Sus	1,020		2,923		523		732	A	830	A	846		968
Sus-Sus	1,125		3,500		628		525	B	715	AB	698		888
*Fertilizer*													
None	1,070		1,879	b	316	b	453	b	560	b	700	b	916
Manure	1,057		5,716	a	1,198	a	690	a	931	a	887	a	1,053
PK	1,095		1,883	b	237	b	424	b	599	b	700	b	849
ANOVA (*F*-value)													
Crop (C)	0.30		0.11		0.49		24.39	**	4.83	*	0.70		0.33
Site (S) × C	0.38		0.62		0.72		0.30		2.53		0.31		0.68
Fertilizer (F)	0.34		28.23	**	70.73	**	13.08	**	24.70	**	3.72	*	2.32
S × F	1.92		0.85		0.15		0.30		1.01		1.48		0.17
F × C	0.64		1.42		2.34		2.44		2.55		0.35		1.52
S × F × C	3.62	*	0.52		0.47		0.52		1.06		0.84		0.35

**Notes:** ^a^Pi, P45d, Pm, and Pf are mean population densities (nematodes/100 cm^3^ soil) prior to applying fertilizer or planting, 45 d after planting, at midseason (about 2 months after planting), and at harvest, respectively; ^b^ * and ** represent significant effects at *P* ⩽ 0.05 and *P* ⩽ 0.01. Values followed by different letters in the same column within the same treatment are significantly different according to Fischer’s least significance test at *P* < 0.05; ^c^crop sequence treatments of SCN-susceptible soybean (Sus), SCN-resistant soybean (Res), or corn for 2009 crop-2010 crop.

Herbivore abundances were greater in susceptible soybean than corn or resistant soybean in Midseason 2009, but greater in corn than the other crop sequences in Fall 2010 (Table 2). Throughout most of the trial, fertilizer treatments did not affect herbivore abundances, but abundances were greater without fertilizer at the end of the trial (Table 2). At 45 DAP 2009, fertilizer effects varied by site (Table 2) and manure decreased herbivore abundances compared with no fertilizer or PK at the suppressive site only (data not shown). Similarly, in midseason 2009, crop sequence effects varied by site (Table 2), and PK increased herbivore abundances compared with no fertilizer at the suppressive site only (data not shown).

**Table 2:** Effects of crop sequence and fertilizer on herbivore abundances by sampling date combined across SCN-conducive and suppressive sites.^a^

	2009							2010				
	Pi	^b^	P45d		Pm		Pf	Pi		Pm	Pf	
*Crop sequence* ^*c*^												
Corn-Sus	1,173		1,050		274	B	520	580	B	737	1,256	A
Res-Sus	1,203		1,225		280	B	470	510	B	668	850	B
Sus-Sus	1,186		1,265		361	A	604	783	A	710	728	B
*Fertilizer*												
None	1,193	ab	1,180	a	306		623	614		770	1,128	a
Manure	1,024	b	958	b	299		478	628		655	848	b
PK	1,345	ab	1,401	a	309		493	632		690	859	b
ANOVA (*F*-value)												
Crop (C)	0.36		0.95		4.54	*	2.74	10.98	**	0.08	8.05	**
Site (S) × C	1.18		0.18		0.37		1.31	5.65	*	0.11	0.54	
Fertilizer (F)	3.87	*	9.02	**	0.16		1.68	0.05		0.29	3.39	*
S × F	2.83		5.44	**	1.18		0.11	2.37		0.10	0.64	
F × C	0.45		0.75		4.23	**	0.93	0.51		1.40	0.72	
S × F × C	0.87		0.34		0.31		1.11	0.58		0.39	0.07	

**Notes:** ^a^Pi, P45d, Pm, and Pf are mean population densities (nematodes/100 cm^3^ soil) prior to applying fertilizer or planting, 45 d after planting, at midseason (about 2 months after planting), and at harvest, respectively; ^b^ * and ** represent significant effects at *P* ⩽ 0.05 and *P* ⩽ 0.01. Values followed by different letters in the same column within the same treatment are significantly different according to Fischer’s least significance test at *P* < 0.05; ^c^Crop sequence treatments of SCN-susceptible soybean (Sus), SCN-resistant soybean (Res), or corn for 2009 crop-2010 crop.

Fungivore abundances were not consistently affected by crop sequence or fertilizer treatments (Table 3). Abundances were greater in resistant soybean than corn in Fall 2009, but were greater following corn than soybean by the end of 2010 (Midseason and Fall). Fungivore abundances were greater in manure than PK in Midseason 2009, but were not affected by fertilizer in any other season.

**Table 3:** Effects of crop sequence and fertilizer on fungivore abundances by sampling date combined across SCN-conducive and suppressive sites.^a^

	2009							2010				
	Pi	^b^	P45d	Pm		Pf		Pi	Pm		Pf	
*Crop sequence* ^*c*^												
Corn-Sus	582		904	168		143	B	208	371	A	349	A
Res-Sus	513		653	145		286	A	238	241	B	285	AB
Sus-Sus	503		665	126		197	AB	188	207	B	245	B
*Fertilizer*												
None	553		782	151	ab	210		199	252		293	
Manure	513		611	163	a	190		237	303		291	
PK	531		829	125	b	226		199	265		293	
*ANOVA (F-value)*												
Crop (C)	1.06		1.87	2.68		3.85	*	2.61	6.21	*	8.43	**
Site (S) × C	2.07		0.96	0.72		0.32		3.59	0.33		0.20	
Fertilizer (F)	0.59		2.10	3.78	*	0.05		2.24	0.21		0.68	
S × F	1.14		2.04	0.01		1.05		2.64	1.83		2.66	
F × C	2.31		0.25	0.88		1.39		2.47	1.81		1.33	
S × F × C	1.87		0.64	0.69		0.32		1.44	1.29		1.22	

**Notes:** ^a^Pi, P45d, Pm, and Pf are mean population densities (nematodes/100 cm^3^ soil) prior to applying fertilizer or planting, 45 d after planting, at midseason (about 2 months after planting), and at harvest, respectively; ^b^ * and ** represent significant effects at *P* ⩽ 0.05 and *P* ⩽ 0.01. Values followed by different letters in the same column within the same treatment are significantly different according to Fischer’s least significance test at *P* < 0.05; ^c^Crop sequence treatments of SCN-susceptible soybean (Sus), SCN-resistant soybean (Res), or corn for 2009 crop-2010 crop.

Fertilizer effects on omnivore-predator abundances varied by crop at Spring 2010 (data not shown). Manure increased omnivore-predator abundances compared with PK or no fertilizer in resistant soybean, but fertilizers did not affect abundances in corn or susceptible soybean (data not shown). Neither fertilizer nor crop sequence treatments affected omnivore-predator abundances at any other time. Average omnivore-predator abundance was 28 nematodes 100 cm^−3^ soil.

### Soil food web indices

From Midseason 2009 through Fall 2010, FBPP was significantly affected by crop sequence treatments (Table 4). Resistant soybean generally increased values compared with other treatments, but this varied by season (Table 4). In Midseason 2009, crop sequence effects varied by site (Table 4), and susceptible soybean increased values compared with corn or resistant soybean at the suppressive site only (data not shown). Throughout most of the study, FBPP values were greater for manure than PK or no fertilizer (Table 4).

**Table 4:** Effects of crop sequence and fertilizer on FBPP (fungivores plus bacterivores divided by herbivores) by sampling date combined across SCN-conducive and suppressive sites.^a^

	2009								2010					
	Vi	^b^	V45d		Vm		Vf		Vi		Vm		Vf	
*Crop sequence* ^*c*^														
Corn-Sus	1.8		6.6		5.1	A	1.3	B	1.6	B	1.7	A	1.3	B
Res-Sus	1.6		3.7		3.9	AB	2.9	A	2.6	A	1.8	A	1.8	A
Sus-Sus	1.6		9.7		2.7	B	1.3	B	1.2	B	1.4	B	1.8	A
*Fertilizer*														
None	1.8		2.6	b	2.4	b	1.5	b	1.5	b	1.5		1.4	b
Manure	1.8		15.2	a	7.2	a	2.3	a	2.4	a	1.9		1.9	a
PK	1.5		2.1	b	2.2	b	1.7	ab	1.5	b	1.5		1.6	ab
*ANOVA (F-value)*														
Crop (C)	0.81		1.17		7.77	**	11.53	**	10.73	**	3.79	*	4.40	*
Site (S) × C	0.91		1.06		4.58	*	3.62		1.36		0.14		0.90	
Fertilizer (F)	2.04		7.28	**	32.70	**	3.39	*	15.66	**	2.83		5.05	*
S × F	0.46		4.53	*	9.71	**	0.30		2.51		1.80		1.60	
F × C	0.28		1.51		1.86		0.83		1.82		0.63		0.78	
S × F × C	0.22		1.28		1.15		0.86		0.51		0.59		0.75	

**Notes:** ^a^Vi, V45d, Vm, and Vf are values prior to applying fertilizer or planting, 45 d after planting, at midseason (about 2 months after planting), and at harvest, respectively; ^b^ * and ** represent significant effects at *P* ⩽ 0.05 and *P* ⩽ 0.01. Values followed by different letters in the same column within the same treatment are significantly different according to Fischer’s least significance test at *P*<0.05; ^c^Crop sequence treatments of SCN-susceptible soybean (Sus), SCN-resistant soybean (Res), or corn for 2009 crop-2010 crop.

Channel index values were greater following corn than soybean in Midseason 2010; however, they were not affected by crop sequences at any other time (Table 5). Manure decreased channel index values compared with PK or no fertilizer throughout most of the study (Table 5). In contrast, enrichment index values were greater in manure than PK or no fertilizer throughout most of the study (Table 6). Resistant soybean increased enrichment index values compared with corn or susceptible soybean in Midseason 2009, but values were not affected by crop sequences during most of the study (Table 6).

**Table 5:** Effects of crop sequence and fertilizer on Channel Index by sampling date combined across SCN-conducive and suppressive sites.^a^

	2009								2010					
	Vi	^b^	V45d		Vm		Vf		Vi		Vm		Vf	
*Crop sequence* ^*c*^														
Corn-Sus	59		21		21		29		19		20	A	14	
Res-Sus	49		18		20		27		13		10	B	12	
Sus-Sus	54		18		21		24		13		11	B	11	
*Fertilizer*														
None	59		25	a	28	a	29	b	18	a	14		13	ab
Manure	54		5	b	6	b	13	c	10	b	12		10	b
PK	50		27	a	27	a	38	a	16	a	16		15	a
*ANOVA (F-value)*														
Crop (C)	2.59		0.44		0.02		0.57		2.36		5.41	*	0.72	
Site (S) × C	0.84		0.02		0.04		2.42		0.17		0.52		0.28	
Fertilizer (F)	2.33		28.51	**	32.27	**	29.96	**	13.63	**	1.46		4.20	*
S × F	2.14		1.25		3.21	*	7.63	**	0.08		0.00		0.48	
F × C	2.79	*	2.19		0.44		0.31		2.35		0.30		0.22	
S × F × C	1.53		1.26		0.46		1.30		0.37		0.49		0.38	

**Notes:** ^a^Vi, V45d, Vm, and Vf are values prior to applying fertilizer or planting, 45 d after planting, at midseason (about 2 months after planting), and at harvest, respectively; ^b^ * and ** represent significant effects at *P* ⩽ 0.05 and *P* ⩽ 0.01. Values followed by different letters in the same column within the same treatment are significantly different according to Fischer’s least significance test at *P* < 0.05; ^c^Crop sequence treatments of SCN-susceptible soybean (Sus), SCN-resistant soybean (Res), or corn for 2009 crop-2010 crop.

**Table 6:** Effects of crop sequence and fertilizer on Enrichment Index by sampling date combined across SCN-conducive and suppressive sites.^a^

	2009							2010						
	Vi	^b^	V45d		Vm		Vf		Vi		Vm		Vf	
*Crop sequence* ^*c*^														
Corn-Sus	40	B	75		75		67		74		77		82	
Res-Sus	45	A	75		77		65		77		84		82	
Sus-Sus	39	B	75		75		66		75		81		81	
*Fertilizer*														
None	40		69	b	75	b	62	b	72	b	80	b	81	b
Manure	41		91	a	95	a	79	a	81	a	84	a	85	a
PK	43		64	b	72	b	56	c	74	b	78	b	79	b
*ANOVA (F-value)*														
Crop (C)	5.45	*	0.02		0.09		0.13		0.51		3.48		0.58	
Site (S) × C	0.29		0.24		0.08		2.44		0.50		0.28		0.17	
Fertilizer (F)	1.20		56.47	**	60.59	**	32.64	**	8.66	**	5.64	**	4.80	*
S × F	3.38	*	1.24		0.49		0.74		0.01		0.53		1.32	
F × C	1.70		2.37		2.67	*	1.44		0.97		0.31		0.64	
S × F × C	1.61		0.88		2.24		2.16		0.94		0.85		0.23	

**Notes:** ^a^Vi, V45d, Vm, and Vf are values prior to applying fertilizer or planting, 45 d after planting, at midseason (about 2 months after planting), and at harvest, respectively; ^b^ * and ** represent significant effects at *P* ⩽ 0.05 and *P* ⩽ 0.01. Values followed by different letters in the same column within the same treatment are significantly different according to Fischer’s least significance test at *P* < 0.05; ^c^Crop sequence treatments of SCN-susceptible soybean (Sus), SCN-resistant soybean (Res), or corn for 2009 crop-2010 crop.

Structure index values were significantly greater in corn than soybean in Fall 2009, but were not affected by crop sequence or fertilizer treatments at any other time (data not shown). Structure index values were small across the study, averaging 12 across all sites, treatments, and sampling dates.

Diversity, based on the Shannon–Weaver diversity index, was not affected by crop sequences (Table 7). At 45 DAP and Midseason 2009, manure decreased diversity compared with PK or no fertilizer treatments (Table 7). In Spring 2010, fertilizer effects varied by crop (Table 7). In Spring 2010, there were no fertilizer effects following resistant soybean, following susceptible soybean values were greater in PK than manure, and following corn values were lesser with no fertilizer than manure or PK (data not shown). At Midseason 2010, fertilizer effects varied by site (Table 7) and manure increased diversity values compared with PK or no fertilizer at the conducive site, but there were no fertilizer effects at the suppressive site.

**Table 7:** Effects of crop sequence and fertilizer on Shannon–Weaver Diversity Index by sampling date combined across SCN-conducive and suppressive sites.^a^

	2009							2010				
	Vi	^b^	V45d		Vm		Vf	Vi		Vm		Vf
*Crop sequence* ^*c*^												
Corn-Sus	1.97		1.64		1.65		1.84	2.00		2.07		2.11
Res-Sus	1.99		1.71		1.72		1.91	1.96		2.00		2.03
Sus-Sus	2.00		1.61		1.69		1.95	1.89		2.05		2.14
*Fertilizer*												
None	1.98		1.82	a	1.86	a	1.90	1.93	b	2.04	b	2.05
Manure	1.99		1.25	b	1.34	b	1.87	1.88	b	1.97	b	2.09
PK	1.99		1.89	a	1.86	a	1.92	2.03	a	2.11	a	2.14
*ANOVA (F-value)*												
Crop (C)	0.17		0.62		0.22		2.95	2.22		1.14		0.99
Site (S) × C	0.46		0.73		0.09		0.05	2.00		2.01		0.00
Fertilizer (F)	0.09		33.91	**	36.37	**	0.77	7.88	**	7.07	**	1.95
S × F	1.66		5.27	**	6.95	**	2.67	1.52		4.16	*	1.91
F × C	0.09		1.88		2.78	*	1.57	3.52	*	1.28		0.77
S × F × C	0.64		0.11		0.60		1.21	1.43		1.26		0.27

**Notes:** ^a^ Vi, V45d, Vm, and Vf are values prior to applying fertilizer or planting, 45 d after planting, at midseason (about 2 months after planting), and at harvest, respectively; ^b^ * and ** represent significant effects at *P* ⩽ 0.05 and *P* ⩽ 0.01. Values followed by different letters in the same column within the same treatment are significantly different according to Fischer’s least significance test at *P* < 0.05; ^c^ Crop sequence treatments of SCN-susceptible soybean (Sus), SCN-resistant soybean (Res), or corn for 2009 crop-2010 crop.

## Discussion

Our results suggested that swine manure application enriches the nematode community in ways that conventional PK fertilizer or no fertilizer does not. In contrast, conventional PK fertilizer did not shift nematode community composition relative to unfertilized control. Swine manure application strongly and consistently increased extreme enrichment opportunist nematodes – c-p1 bacterivores – that thrive on abundant, simple resources and rapidly colonize an environment under those conditions. These enrichment opportunist nematodes provide important services such as nutrient cycling that mineralize nutrients, particularly P and K, into plant-available forms (Holajjer et al., 2016; Trap et al., 2016). This suggests manure application enriched the soil ecosystem compared with conventional PK fertilizer or no fertilizer, an assertion that was reinforced by a consistent increase in enrichment index values. The soil food web enrichment driven by manure application was likely due to the greater amount organic matter and nitrogen in swine manure compared with conventional fertilizer (Hernandez et al., 2007) providing resources for colonizer organisms including bacteria and bacterivores, particularly organic carbon sources, whereas conventional fertilizers contain few or no carbon sources (Wolf and Wagner, 2005).

In the two years of this study, the influx of resources from manure application affected only nematodes that are highly adapted enrichment opportunists. Manure application generally did not affect c-p2 bacterivores or fungivores, which are also enrichment opportunists (Bongers, 1990). Omnivores and predators, which are persister strategists, were not affected by manure or conventional fertilizer application and were not very abundant overall. Similarly, neither manure nor fertilizer application affected community structure – the relative number of trophic links in a system – based on structure index. Manure was only applied once in this study and impacts on the soil food web were observed for two years after application, which is a relatively short time period. Perhaps manure application would increase abundances of organisms higher in the food chain if given further time for resources to flow through the food web, and potentially repeated manure applications, through bottom-up effects. Further research would be needed to investigate this.

Similar studies have generally found swine manure products (Bulluck et al., 2002; Mahran et al., 2009; Renco and Kovacik, 2012; Grabau et al., 2018) or other organic fertilizer amendments (Briar et al., 2007; Hu and Cao, 2008; Overstreet et al., 2010; Treonis et al., 2010) enrich the soil food web relative to no fertilizer application or conventional fertilizers. A few studies have found that conventional fertilizers enrich the soil community and increase bacterivore abundances compared with unfertilized land in a similar manner to manure or other organic amendments (Okada and Harada, 2007; Zhang et al., 2016). Community maturity or structure was not measured in many similar studies, but when it has been measured, organic fertilizer application either had no effect on maturity (Briar et al., 2007; Treonis et al., 2010) or decreased maturity (Bulluck et al., 2002).

In many similar studies, manure application has generally increased abundances of all enrichment opportunists, including fungivores (Bulluck et al., 2002; Briar et al., 2007; Hu and Cao, 2008; Renco and Kovacik, 2012), and both c-p1 and c-p2 bacterivores when measured (Liang et al., 2009). In one study, swine manure application had a similar impact as that observed in this study, increasing bacterivore abundances consistently but generally not affecting other trophic groups (Grabau et al., 2018). Differences in source material for the organic fertilizers used in this study compared with many previous studies may partially account for these differences since fungi and fungivores are more abundant in soils amended with organic materials of greater C:N ratios and more recalcitrant organic material than bacteria and bacterivores (Broder and Wagner, 1988; Wagner and Broder, 1993). In this study and Grabau et al. (2018), anaerobically digested swine manure – not mixed with any recalcitrant organic material – was applied. The other previous studies used slightly different material such as raw, undigested swine manure (Bulluck et al., 2002); swine manure that was composted (Liang et al., 2009) or mixed with sawdust (Renco and Kovacik, 2012); or cow manure composted with straw (Briar et al., 2007; Hu and Cao, 2008). Composting swine or cow manure changes the chemical and nutrient profile of these products, notably increasing the C:N ratio compared with raw or anaerobically digested manure (Troy et al., 2012; Qian et al., 2014). Raw swine and cow manure also differ slightly in their chemical and nutrient profiles (Qian et al., 2014).

Manure application somewhat decreased overall herbivore abundance at 45 DAP suggesting swine manure may have had some nematicidal effects on herbivores abundances. However, swine manure had little effect on abundances of the economically important plant-parasitic nematodes found at the site, primarily SCN, although it alleviated SCN damage to soybean at the SCN-conducive site as reported in Bao et al., (2013) study.

The increase in enrichment opportunists, but not other trophic groups, is indicative of broader changes in community structure and the flow of nutrients through the soil food web with the addition of manure. Manure application shifted decomposition pathways toward bacterial channels and away from fungal channels based on the channel index which is similar to the results in other studies (Liang et al., 2009). Similarly, manure application dramatically increased FBPP – the ratio of fungivore and bacterivores to herbivores. Greater FBPP values are interpreted to indicate greater beneficial services relative to detrimental effects from the nematode community (Wasilewska, 1989) since fungivores and bacterivores provide beneficial services, such as nutrient cycling, while herbivores are detrimental for crop production. Manure application decreased community diversity because that treatment stimulated population growth of a few genera of extreme colonizers to the exclusion of other nematodes. A number of nematode community indices have been proposed (Bongers, 1990; Ferris et al., 2001), and it is relatively infrequent for FBPP to be included in nematode community studies (Bulluck et al., 2002; Hu and Cao, 2008; Renco and Kovacik, 2012). In this study, FBPP was as sensitive as or more sensitive than many other indices including enrichment, channel, and structure index and provided distinct information about nematode community structure.

The influence of manure application on colonizer nematode abundances and soil food web structure continued over a year following fertilizer applications indicating manure application had residual effects on soil biology. These residual effects are likely due to a combination of continued influx of resources from continued decomposition of organic matter in manure (Diaz et al., 2012) and residual differences in nematode abundances among treatments from the fertilizer application event at the start of the study. In general, nematode community responses to fertilizer application were relatively similar among sites and crop sequences in this study.

As stated in the introduction, a long-term goal of this study was to identify strategies that improve sustainability (long-term productivity while conserving natural resources) of corn-soybean systems, particularly biological aspects of soil quality. Manure application clearly affected the nematode community and soil ecology, and some of these effects were positive such as stimulating populations of bacterivores that are very important for nutrient cycling (Trap et al., 2016), and increasing the ratio of beneficial to detrimental nematodes. Some of these effects are generally considered negative such as decreasing diversity and maturity which may reflect a community that is less resilient and resistant. Crop productivity, a key component of sustainability, was also improved with manure application (Bao et al., 2013), although more work would be needed to quantify if increased productivity was the result of increased nutrient input, changes in soil biology or a combination of factors. Further research and analysis would be needed to fully determine if the changes in soil ecology observed in this study are positive or negative and the degree to which manure application is a sustainable practice, particularly over a longer period of continuous practice.

Our results suggested that short-term crop sequences have a much less substantial and consistent influence on nematode community composition than swine manure application. Soybean, particularly resistant soybean, tended to encourage bacterivore population growth relative to corn, but only near the end of the first growing season and beginning of the second growing season. Conversely, corn tended to encourage fungivore population growth relative to soybean late in the year following production of these crops, although this trend was reversed at the end of the first growing season. In Midseason 2010, decomposition pathways were more fungal-dominated than bacterial-dominated in corn relative to soybean production, which also reflects these differences.

In a long-term corn-soybean rotation study in Minnesota near the site of the present study, corn also favored fungivores while soybean favored bacterivores, but it often took multiple growing seasons for these differences between cropping environments to be detectable (Grabau and Chen, 2016c). That trend is consistent with the relatively weak cropping effects in this study following only a single year of different crops. Differences in crop residue composition (Broder and Wagner, 1988; Halvorson and Schlegel, 2012), crop nutrient uptake (Halvorson and Schlegel, 2012), or root exudate profiles (Arafat et al., 2017) between corn and soybean may contribute to differences in nematode community composition in the presence of these crops (Grabau and Chen, 2016c). Other studies also suggest that different crops favor different groups of organisms resulting in varied soil community composition, particularly after cropping systems are implemented for a number of years (Briar et al., 2012; Djigal et al., 2012).

Crop sequences also affected total herbivore abundances during portions of the study with abundances greater under corn or susceptible soybean variously during the study. Based on the results reported in Bao et al. (2013) study, spiral nematode and SCN J2 abundances generally drove these trends as SCN J2 abundances were consistently greater in susceptible soybean rotation and spiral nematode abundances were greater in corn-soybean rotation than soybean toward the end of each year. Because overall herbivore populations were generally lower in resistant soybean than one or both of the other rotations, services provided by the nematode community were skewed toward beneficial and away from detrimental based on FBPP. This is similar to results of other studies (Grabau and Chen, 2016c).

Finally, our results suggested that short-term crop sequences and fertilizer application do not have an interactive effect on the nematode community. This implies that differences in nutrient uptake of fertilizer, soil food web composition, and other factors between corn and soybean did not alter the influence of fertilizer application on the nematode between these crops, at least in the short term. As discussed for fertilizer and crop sequence main effects, the lack of interaction may be due to the short-term nature of both the crop sequences and fertilizer application in this study. A hypothesis to investigate in the future is that long-term combinations of crop sequences and manure application exert a stronger influence on the nematode community than either practice on its own.

In conclusion, short-term crop sequences and fertilizer application did not have an interactive effect on the nematode community. Swine manure enhanced populations of extreme enrichment opportunist nematodes and enriched the soil food web relative to no fertilizer or conventional PK fertilizer. Manure application did not affect other groups of beneficial nematodes and decreased soil food web diversity, so impacts were a mixture of positive and negative. The nematode community was similar without fertilizer and with conventional PK fertilizer. Crop sequences drove some changes in soil ecology, but crops had fewer short-term impacts than manure application.
